# Biomechanical comparison between unilateral and bilateral percutaneous vertebroplasty for osteoporotic vertebral compression fractures: A finite element analysis

**DOI:** 10.3389/fbioe.2022.978917

**Published:** 2022-09-08

**Authors:** Haowen Dai, Yang Liu, Qing Han, Aobo Zhang, Hao Chen, Yang Qu, Jincheng Wang, Jianwu Zhao

**Affiliations:** Department of Orthopedics, The Second Hospital of Jilin University, Changchun, China

**Keywords:** vertebroplasty, osteoporotic vertebral compression fracture, finite element analysis, bone cement, biomechanics

## Abstract

**Background and objective:** The osteoporotic vertebral compression fracture (OVCF) has an incidence of 7.8/1000 person-years at 55–65 years. At 75 years or older, the incidence increases to 19.6/1000 person-years in females and 5.2–9.3/1000 person-years in males. To solve this problem, percutaneous vertebroplasty (PVP) was developed in recent years and has been widely used in clinical practice to treat OVCF. Are the clinical effects of unilateral percutaneous vertebroplasty (UPVP) and bilateral percutaneous vertebroplasty (BPVP) the same? The purpose of this study was to compare biomechanical differences between UPVP and BPVP using finite element analysis.

**Materials and methods:** The heterogeneous assignment finite element (FE) model of T11-L1 was constructed and validated. A compression fracture of the vertebral body was performed at T12. UPVP and BPVP were simulated by the difference in the distribution of bone cement in T12. Stress distributions and maximum von Mises stresses of vertebrae and intervertebral discs were compared. The rate of change of maximum displacement between UPVP and BPVP was evaluated.

**Results:** There were no obvious high-stress concentration regions on the anterior and middle columns of the T12 vertebral body in BPVP. Compared with UPVP, the maximum stress on T11 in BPVP was lower under left/right lateral bending, and the maximum stress on L1 was lower under all loading conditions. For the T12-L1 intervertebral disc, the maximum stress of BPVP was less than that of UPVP. The maximum displacement of T12 after BPVP was less than that after UPVP under the six loading conditions.

**Conclusion:** BPVP could balance the stress of the vertebral body, reduce the maximum stress of the intervertebral disc, and offer advantages in terms of stability compared with UPVP. In summary, BPVP could reduce the incidence of postoperative complications and provide promising clinical effects for patients.

## Introduction

Osteoporosis (OP) is a systemic metabolic disease characterized by reduced bone mass and easily fractured bones, which causes approximately 8.9 million fractures worldwide each year, with an average of one every 3 s (Peng et al., 2018; Lin et al., 2015). The osteoporotic vertebral compression fracture (OVCF) is the most common type of OP-related fracture (Yang et al., 2016b). OVCF has an incidence of 7.8/1000 person-years at 55–65 years. At 75 years or older, the incidence increases to 19.6/1000 person-years in females and 5.2–9.3/1000 person-years in males (Liu et al., 2021). Nowadays, the increasing incidence of OVCF has imposed an economic burden on society and families and has been a major global health problem (Zuo X. H. et al., 2021; Badilatti et al., 2015). The quality of life can be deteriorated by OVCF, which is associated with acute or chronic back pain, functional limitations of the spine, vertebral height (VH) loss, and kyphotic deformity (Zhao et al., 2018; Distefano et al., 2021).

To solve this problem, percutaneous vertebroplasty (PVP), a minimally invasive surgical technique, was developed in recent years. This technique can quickly stabilize the fracture, relieve pain, and reinforce the anterior column through the percutaneous injection of bone cement into the fractured vertebral body (Tsoumakidou et al., 2017; Cianfoni et al., 2019a; Cianfoni et al., 2019b; Hirsch et al., 2021). Meanwhile, PVP could improve the effectiveness of OVCF treatment (Kim et al., 2012). Up to 90% of patients experience immediate and significant pain relief with PVP (Chiu et al., 2021).

Currently, PVP has been widely used in clinical practice to treat OVCF (Buchbinder et al., 2018) and can be divided into unilateral percutaneous vertebroplasty (UPVP) and bilateral percutaneous vertebroplasty (BPVP) (Cheng et al., 2016). Although both methods provide significant pain relief, their postoperative complications can adversely affect patients’ long-term quality of life. Thus, it has remained controversial which PVP approach has a more satisfactory clinical effect (Teles and Mattei, 2015). Some studies comparing UPVP and BPVP were only based on a systematic review and meta-analysis (Huang et al., 2014; Sun and Li, 2016; Cheng et al., 2016) but with little evidence on biomechanics. Comparisons based on biomechanics would be clinically relevant because this might help the surgeon decide which approach could reduce the incidence of postoperative complications of PVP and provide more promising clinical results. Therefore, in this study, a finite-element analysis was used to figure out the biomechanical differences between BPVP and UPVP and provide guidance for clinical application.

## Materials and methods

### The construction of T11-L1 finite element model

A three-dimensional FE model of T11-L1 was first reconstructed using Mimics 21.0 (Materialise, Leuven, Belgium) based on computed tomography (CT) scans with 0.2-mm intervals of a normal male adult without any lumbar disease (Widmer Soyka et al., 2016). This study was approved by the Ethics Committee of the Second Hospital of Jilin University, and informed consent was obtained from the volunteer. Next, the vertebral bodies of T11-L1 were smoothed and polished to obtain a more precise and smoother 3D surface model in Magics 21.0 (Materialise, Leuven, Belgium). Intervertebral discs and endplates were created in 3-matic 13.0 (Materialise, Leuven, Belgium). Subsequently, these structures were meshed in HyperMesh 20.0 (Altair, California, United States) (Liu et al., 2020).

Three-dimensional solid elements with an isotropic character were used to model the vertebral body, endplate, and intervertebral disc (Pei et al., 2022; Bereczki et al., 2021; Umale et al., 2022). The element types of vertebrae were four-node tetrahedral elements (C3D4). According to the empirical formula of a vertebral body, properties of materials were attached to the finite element models (Rho et al., 1995). The relationship between density and gray scale can be presented as:

$$\rho =1.122*HU+47\left(g/mm^{3}\right)$$ (1)

The relationship between elastic modulus and density is given by:

$$E=0.69\rho^{1.35}\left(Mpa\right)$$ (2)

where *ρ* represents density, HU represents the threshold, and E represents elastic modulus.

**FIGURE 1:**
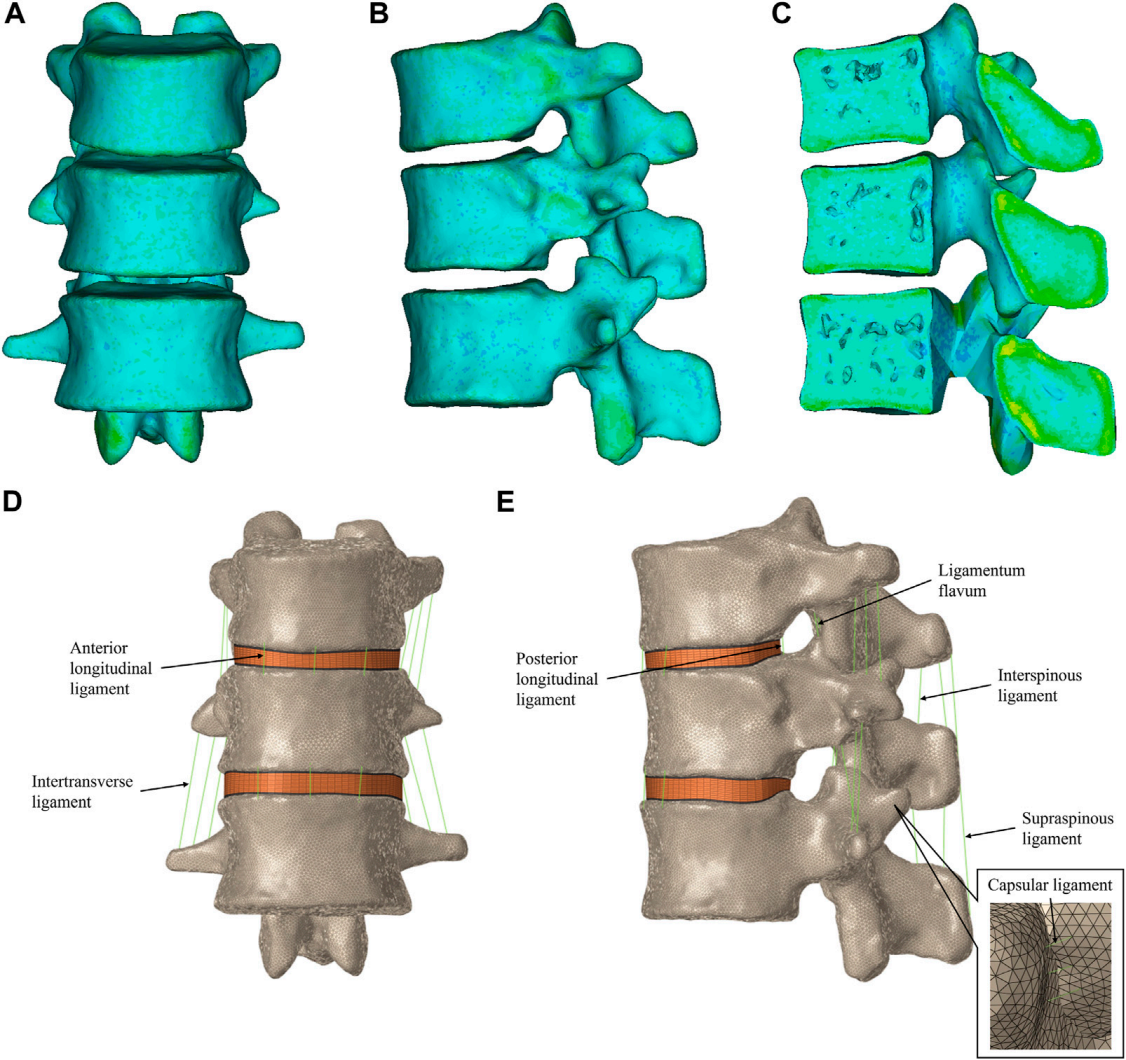
T11-L1 heterogeneous assignment finite element model. **(A)** Front view of the T11-L1 model. **(B)** Left view of the T11-L1 model. **(C)** Section view of the T11-L1 model. T11-L1 heterogeneous assignment finite element model in HyperMesh, including the intervertebral disc and ligament structures. **(D)** Front view of the T11-L1 model. **(E)** Right view of the T11-L1 model.

The materials were divided into ten categories and assigned according to the grayscale of the CT images (Figures 1A–C).

The element types of endplates were eight-node hexahedral elements (C3D8). Each intervertebral disc consisted of a nucleus pulposus surrounded by an annulus ground substance (Guo and Fan, 2018). The annulus was divided into five layers, and the nucleus pulposus was set to occupy approximately 43% of the total surface area of the disc (Polikeit et al., 2003b). The nucleus pulposus and annulus ground substance were meshed by C3D8 with an isotropic and incompressible material (Zhang et al., 2021; Wang and Guo, 2021; Guo and Wang, 2020). The facet joint contacts were defined as surface-to-surface contact elements in combination with a friction coefficient of 0.1 (Zhang et al., 2022; Zhong et al., 2009; Polikeit et al., 2003a), and the contact interfaces of the other components were assigned to be completely bonded.

To get accurate results, the mesh grid of this patient-specific model was verified. In this study, the three-dimensional finite element model of T11-L1 was used for the mesh convergence test. The element size of T11-L1 was varied to yield four different mesh resolutions by keeping the very refined mesh as the reference for comparisons (Table 1). The peak von Mises pressures predicted by cases A–C were compared with those predicted by the reference case. The mesh verification usually achieved with a mesh-independent grid ensures that coarsening of the mesh does not disturb the stress field by more than 5%. Case A was found to be optimal as it required less computational time while maintaining >95% prediction accuracy relative to the reference case model.

**TABLE 1:** The mesh convergence test.

Case (s)	ES^a^(mm)	NOE^a^	% Change in peak von Mises pressure
References	0.5	712,845	-
Case A	0.8	255,400	<5%
Case B	1.2	103,496	>5%
Case C	1.5	67,729	>5%

a ES: element size, NOE: number of elements.

Spinal ligaments [the anterior longitudinal ligament (ALL), posterior longitudinal ligament (PLL), capsular ligament (CL), intertransverse ligament (ITL), ligamentum flavum (LF), interspinous ligament (ISL), and supraspinous ligament (SSL)] were included in the model. These ligaments were modeled with two-node 1D spring elements applied only to the tension force in seven groups (Figures 1D,E) (Tan et al., 2021; Zuo X. et al., 2021; Elmasry et al., 2017). All material properties and element types of the above tissues are listed in Table 2.

**TABLE 2:** Material properties of finite element analysis models.

	Young modulus (MPa)	Poisson ratio	Element type	References
Bone				
Normal vertebral body	0.69ρ^1.35^	0.3	C3D4	Rho et al. (1995)
Osteoporotic vertebral body	(0.69ρ^1.35^)^a^67%	0.3	C3D4	(Polikeit et al., 2003d), (Salvatore et al., 2018)
Normal endplate	1000	0.4	C3D8	Bereczki et al. (2021)
Osteoporotic endplate	670 (67% of normal)	0.4	C3D8	Salvatore et al. (2018)
Intervertebral disc			C3D8	Wang and Guo, (2021)
Annulus ground substance	4.2	0.45		
Nucleus pulposus	1	0.499		
Ligaments			spring	Guo et al. (2020c)
ALL^a^	20	0.3		
PLL^a^	20	0.3		
LF^a^	19.5	0.3		
ITL^a^	59	0.3		
ISL^a^	12	0.3		
SSL^a^	15	0.3		
CL^a^	7.5	0.3		
Bone cement (PMMA^a^)	3000	0.4	C3D4	Yang et al. (2016a)

a ALL, anterior longitudinal ligament; PLL, posterior longitudinal ligament; LF, ligamentum flavum; ITL, intertransverse ligament; ISL, interspinous ligament; SSL, supraspinous ligament; CL, capsular ligament; PMMA, polymethylmethacrylate.

### The simulation of compression fracture

**FIGURE 2:**
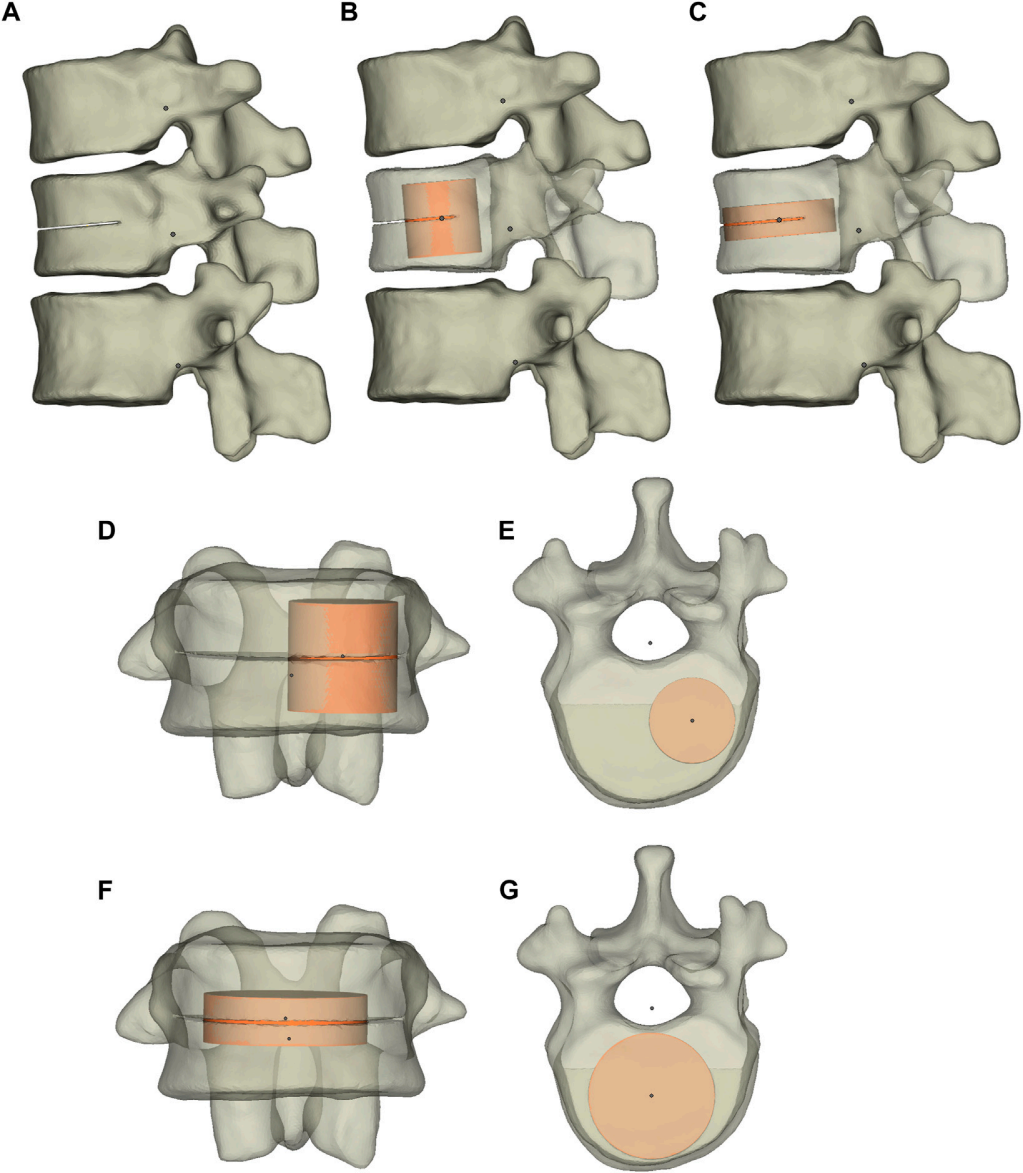
The T11-L1 three-dimensional model and T12 fracture model. The T11-L1 three-dimensional model **(A)** and the T12 vertebral body after UPVP **(B)** and BPVP **(C)**. **(D)** Frontal view of the T12 vertebral body after UPVP. **(E)** Superior view of the T12 vertebral body after UPVP. **(F)** Frontal view of the T12 vertebral body after BPVP. **(G)** Superior view of the T12 vertebral body after BPVP.

The osteoporotic condition was modeled by decreasing the elastic moduli of each category of vertebral bodies by a set amount (Table 2) (Polikeit et al., 2003d; Salvatore et al., 2018; Bereczki et al., 2021; Lu et al., 2022; Zhou et al., 2020; Guo et al., 2020a; Guo et al., 2020b). According to a previously reported simulation method (Liang et al., 2015b), the T12 fracture line models were produced by cutting the vertebral body to produce the 0.5-mm fracture line (Chiang et al., 2009). The fracture line models were performed in Materialise Magics 21.0 (Materialise, Leuven, Belgium). The cleft horizontally penetrated the vertebral body by 22.5 mm through the center of the anterior cortical shell. The cleft size was approximately 22.5, 42.5, and 0.5 mm in depth, width, and height, respectively (Figure 2A).

### The simulation of UPVP and BPVP

The simulation of UPVP and BPVP was performed in Materialise Magics 21.0 (Materialise, Leuven, Belgium). The cement cylinder was vertically implanted into one side of the fractured vertebra to simulate UPVP (Figures 2B,D,E). Another cement cylinder with the same volume was vertically implanted into two sides of the fractured vertebra to simulate BPVP (Figures 2C,F,G). The volume of both cement cylinders was approximately 6.3 ml. The material properties of polymethylmethacrylate (Young’s modulus, 3000 MPa; Poisson ratio, 0.4) were applied to bone cement, and the mechanical properties of the bone cement was assumed to be linear-elastic, isotropic, and homogeneous (Koh et al., 2016). The interface of the vertebral body and bone cement was assigned to be completely bonded.

### Boundary and loading conditions of FE models

**FIGURE 3:**
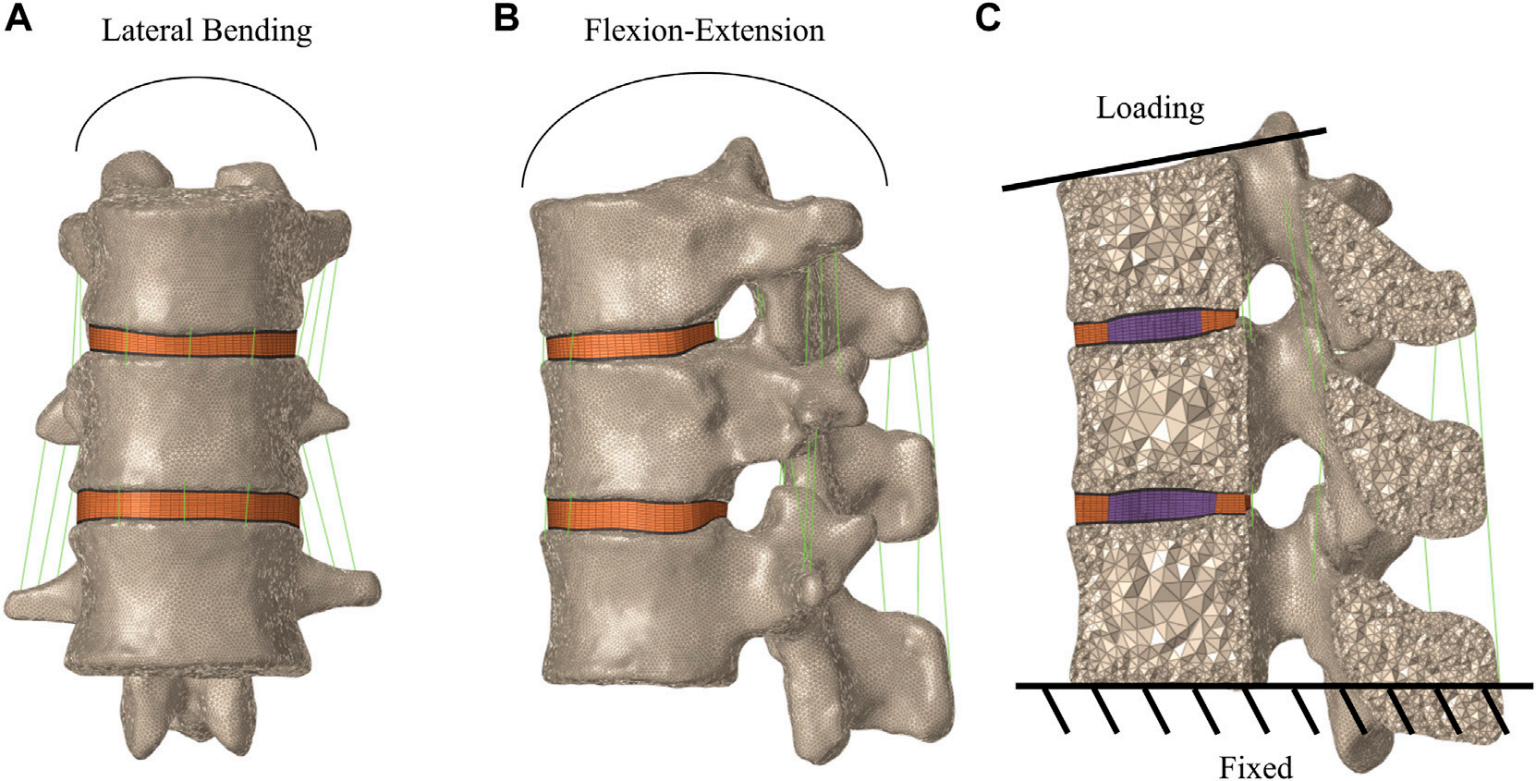
Boundary and loading conditions of finite element models of T11-L1. **(A)** Frontal view of the T11-L1 model. **(B)** Lateral view of the T11-L1 model. **(C)** Loading and the boundary condition.

The lower surface of the L1 vertebral body was fixed in all directions throughout the simulation process. The upper surface of the T11 vertebral body was implemented with a 500 N axial compression load to simulate the weight of the human upper body segment (Chen L. H. et al., 2011; Lo et al., 2008), a pure moment of 7.5 Nm combined with a pre-compressive load of 500 N was implemented for flexion, extension, left/right lateral bending, and left/right axial rotation (Figure 3). The distributions and magnitudes of the von Mises stress on each vertebral body and intervertebral disc were calculated. Moreover, the maximum displacement of the T12 vertebral body was used for analysis. The von Mises stress has been proposed as a parameter of failure criteria for the bone, and maximum displacement has been proposed as a parameter of stability (Polikeit et al., 2003d; Li Q.-l. et al., 2013; Liang et al., 2015a).

## Results

### Validation of T11-L1 thoracolumbar vertebral model

**FIGURE 4:**
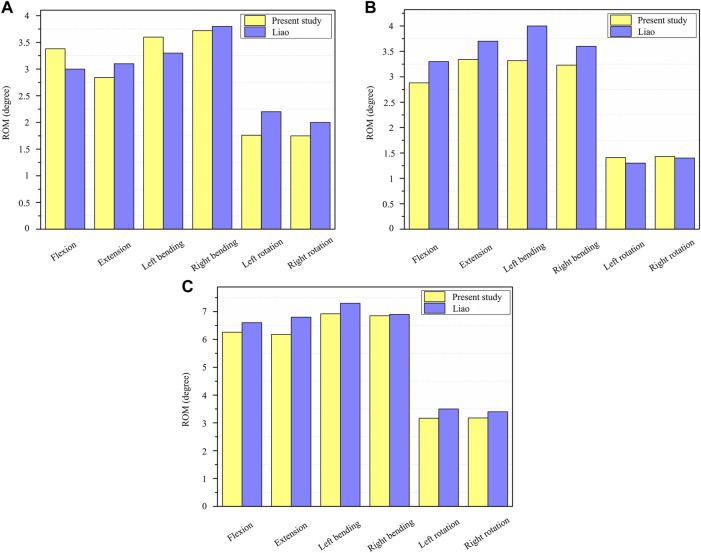
Validation of the T11-L1 thoracolumbar vertebral model. **(A)** The ROM of T11-T12 under six loading conditions; **(B)** The ROM of T12-L1 under six loading conditions; **(C)** The ROM of T11-L1 under six loading conditions.

The current FE model was validated in combined loading of both pure moment and follower load, and the predicted results of range of motion (ROM) was compared with the numerical data obtained under the identical condition to validate the FE model. The present model results were in good agreement with the results obtained from the literature (Liao, 2020), and the detailed model verification results are shown in Table 3; Figure 4.

**TABLE 3:** Comparison of the ROM of T11–T12, T12–L1 and T11-L1 with published study of Liao (Liao, 2020) (unit: degree).

	T11-T12		T12-L1		T11-L1	
	Present study	Liao	Present study	Liao	Present study	Liao
Flexion	3.38	3.00	2.88	3.30	6.26	6.60
Extension	2.84	3.10	3.34	3.70	6.18	6.80
Left bending	3.60	3.30	3.32	4.00	6.92	7.30
Right bending	3.72	3.80	3.23	3.60	6.85	6.90
Left rotation	1.76	2.20	1.41	1.30	3.17	3.50
Right rotation	1.75	2.00	1.43	1.40	3.18	3.40

### The distributions and magnitudes of the von mises stress on T12

**FIGURE 5:**
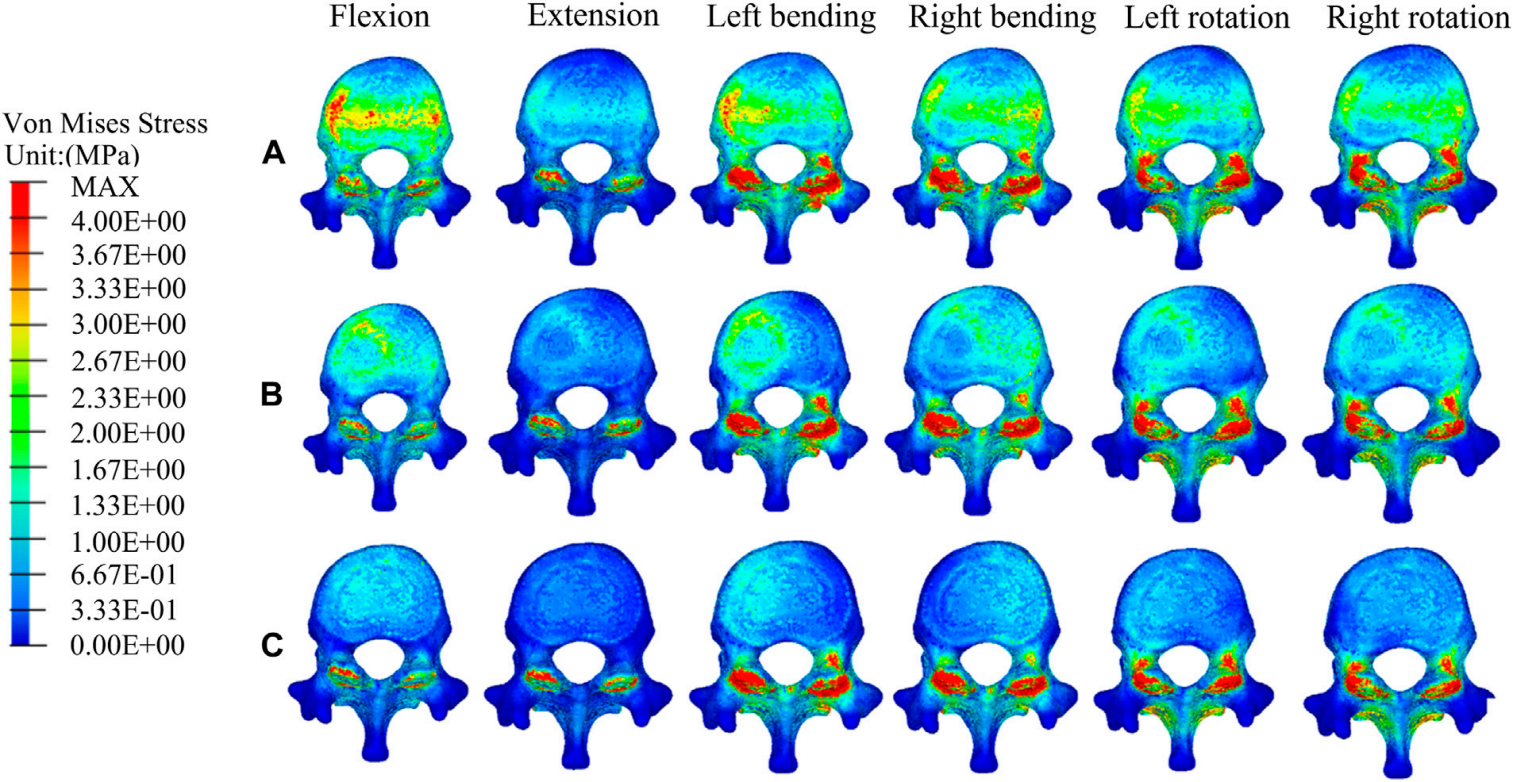
The distributions of the von Mises stress on T12. The distributions of the von Mises stress on T12 in a fractured model **(A)**, UPVP **(B)**, and BPVP **(C)** under flexion, extension, left/right bending, and left/right torsion.

Figure 5 shows the stress distributions on T12 in the fractured model, UPVP, and BPVP. The stress distribution in UPVP was concentrated on the junction of the bone cement and the vertebral body under six loading conditions. However, compared with the fractured model and UPVP, there was no obvious high-stress concentration region on the anterior and middle columns of the T12 vertebral body in BPVP.

**FIGURE 6:**
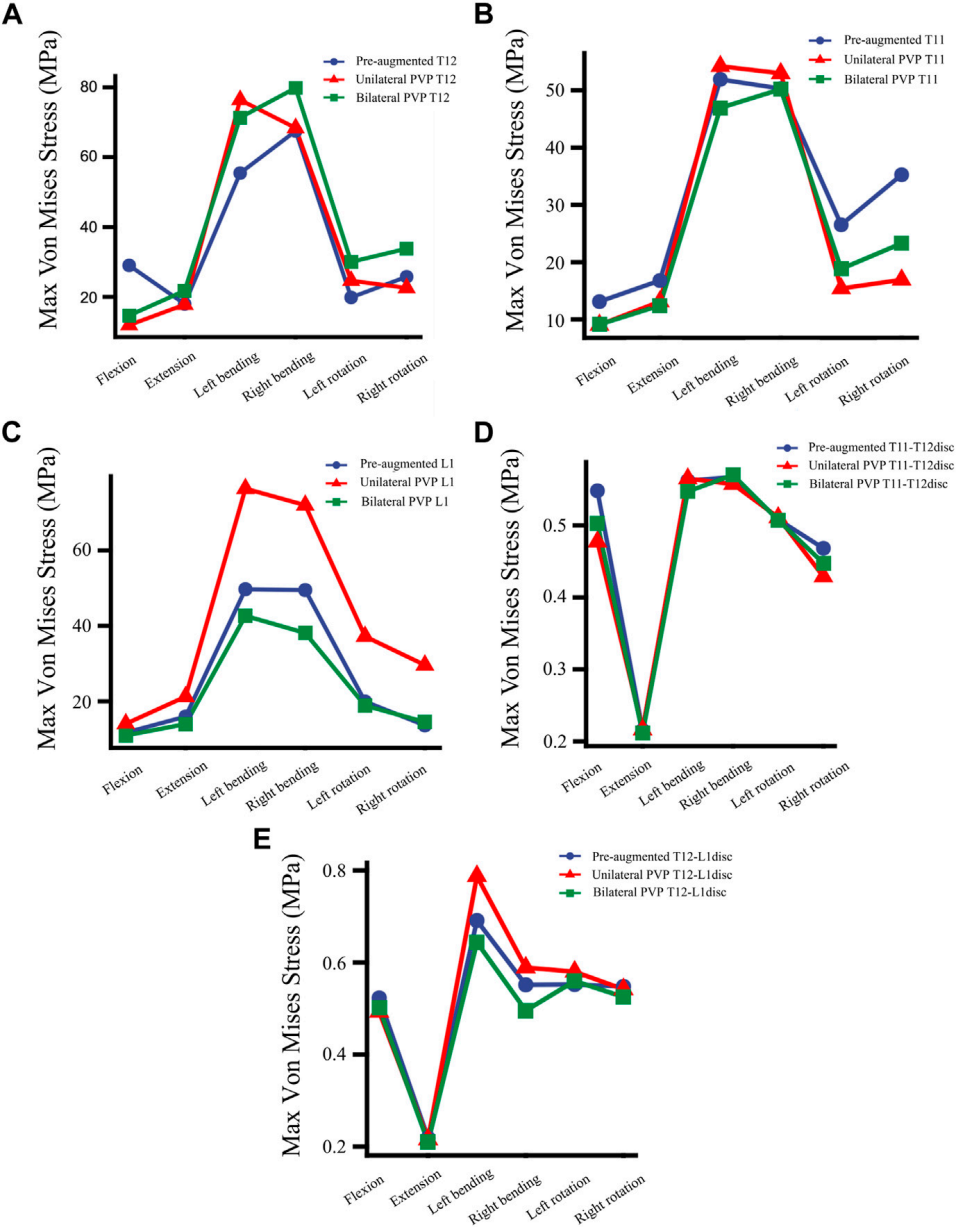
The maximum von Mises stress at vertebrae and discs. **(A)** The maximum von Mises stress at T12 in a fractured model (Pre-augmented), UPVP, and BPVP under flexion, extension, left/right bending, and left/right torsion, **(B)** the maximum von Mises stress at T11 in a fractured model, UPVP, and BPVP under flexion, extension, left/right bending, and left/right torsion, **(C)** the maximum von Mises stress on L1 in a fractured model, UPVP, and BPVP under flexion, extension, left/right bending, and left/right torsion, **(D)** the maximum von Mises stress on the T11-T12disc in a fractured model, UPVP, and BPVP under flexion, extension, left/right bending, and left/right torsion, **(E)** the maximum von Mises stress on the T12-L1disc in the fractured model, UPVP, and BPVP under flexion, extension, left/right bending, and left/right torsion.

Compared with the fractured model, the maximum stress on T12 in UPVP increased under left lateral bending and left axial rotation (Figure 6A). However, it decreased under flexion, extension, right lateral bending, and right axial rotation. Compared with the fractured model, the maximum stress on T12 in BPVP increased under extension, lateral bending, and axial rotation. Compared with UPVP, the maximum stress on T12 in BPVP was higher under six loading conditions, which were 14.69, 21.77, 71.33, 79.81, 30.03, and 33.81 MPa.

### The distributions and magnitudes of the von mises stress on adjacent T11 and L1

**FIGURE 7:**
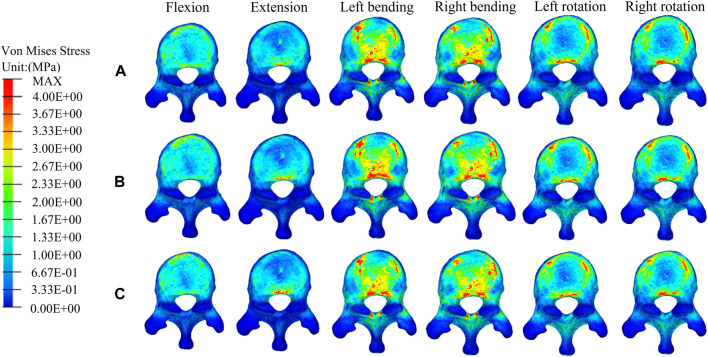
The distributions of the von Mises stress on T11. The distributions of the von Mises stress on T11 in the fractured model **(A)**, UPVP **(B)**, and BPVP **(C)** under flexion, extension, left/right bending, and left/right torsion.

**FIGURE 8:**
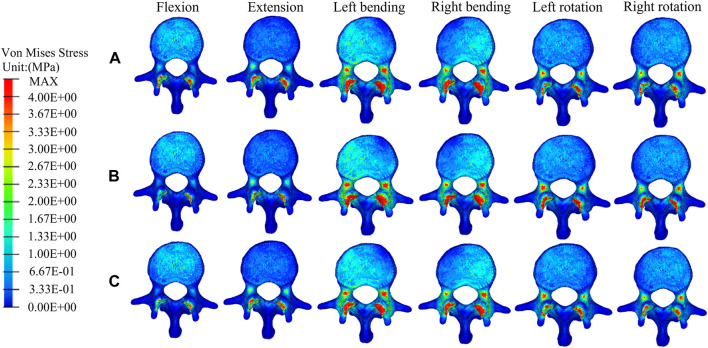
The distributions of the von Mises stress on L1. The distributions of the von Mises stress on L1 in a fractured model **(A)**, UPVP **(B)**, and BPVP **(C)** under flexion, extension, left/right bending, and left/right torsion.

Figures 7, 8 show the stress distributions on the adjacent T11 and L1 in a fractured model, UPVP, and BPVP. Compared with the fractured model, it was unchanged after both UPVP and BPVP under six loading conditions. There was no obvious high-stress concentration region on the anterior and middle columns of the L1 vertebral body in the fractured model, UPVP, and BPVP. However, on the anterior and middle columns of the T11 vertebral body, there were obvious stress concentration regions.

As shown in Figure 6B, the maximum stress on the adjacent T11 of UPVP increased under left/right lateral bending compared with the fractured model. However, it decreased under other loading conditions. The maximum stress on T11 of BPVP decreased under all loading conditions compared to the fractured model. The maximum stress on T11 of UPVP under six loading conditions was 9.044, 13.11, 54.21, 52.97, 15.40, and 16.92 MPa, respectively, and that of BPVP was about 9.127, 12.43, 46.93, 50.19, 18.82, and 23.29 MPa, respectively. Compared with UPVP, the maximum stress was higher under left and right axial rotation, lower under left and right lateral bending, and almost the same under flexion and extension. As shown in Figure 6C, the maximum stress on L1 of BPVP under all loading conditions was the least of the three models (including the fractured model, UPVP, and BPVP).

### The distributions and magnitudes of the von mises stress on T11-T12 disc and T12-L1 disc

**FIGURE 9:**
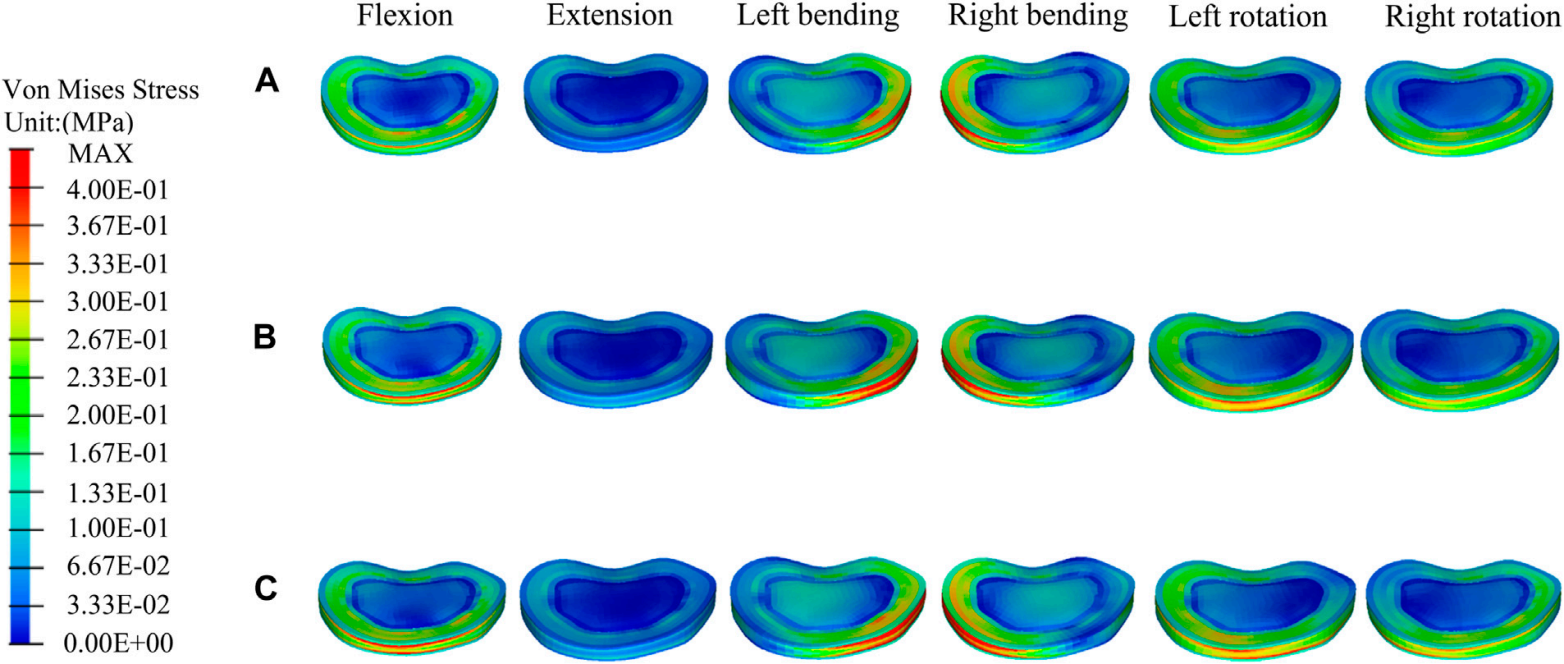
The distributions of the von Mises stress on the T11-T12 disc. The distributions of the von Mises stress on the T11-T12disc in a fractured model **(A)**, UPVP **(B)**, and BPVP **(C)** under flexion, extension, left/right bending, and left/right torsion.

**FIGURE 10:**
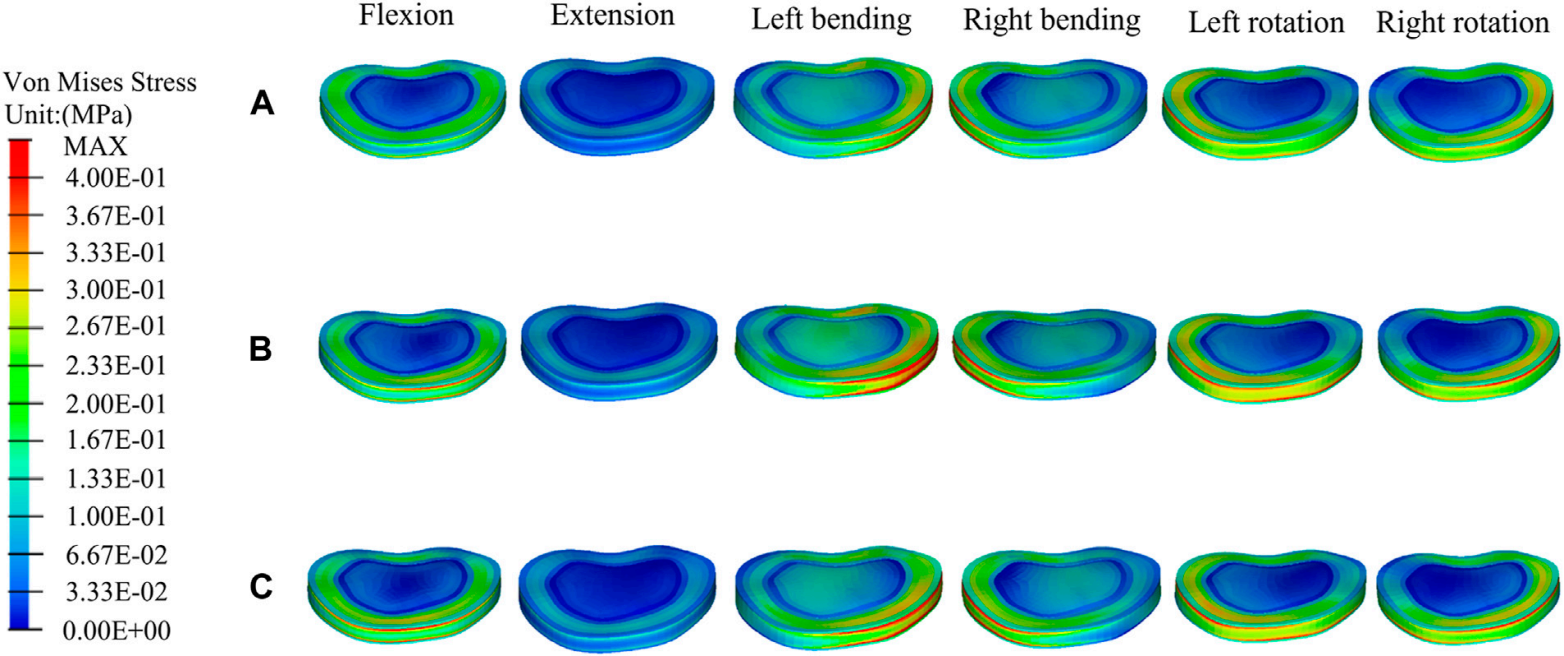
The distributions of the von Mises stress on the T12-L1disc. The distributions of the von Mises stress on the T12-L1disc in a fractured model **(A)**, UPVP **(B)**, and BPVP **(C)** under flexion, extension, left/right bending, and left/right torsion.

The stress distributions at the T11-T12 disc and the T12-L1 disc did not change significantly in the fractured model, UPVP, and BPVP under six conditions (Figures 9, 10).

For the T11-T12 intervertebral disc, the maximum stresses of a fractured model, UPVP, and BPVP had little difference under six loading conditions (Figure 6D). However, for the T12-L1 intervertebral disc, the maximum stress of BPVP was less than those of UPVP and a fractured model (Figure 6E).

### The maximum displacement of T12

**FIGURE 11:**
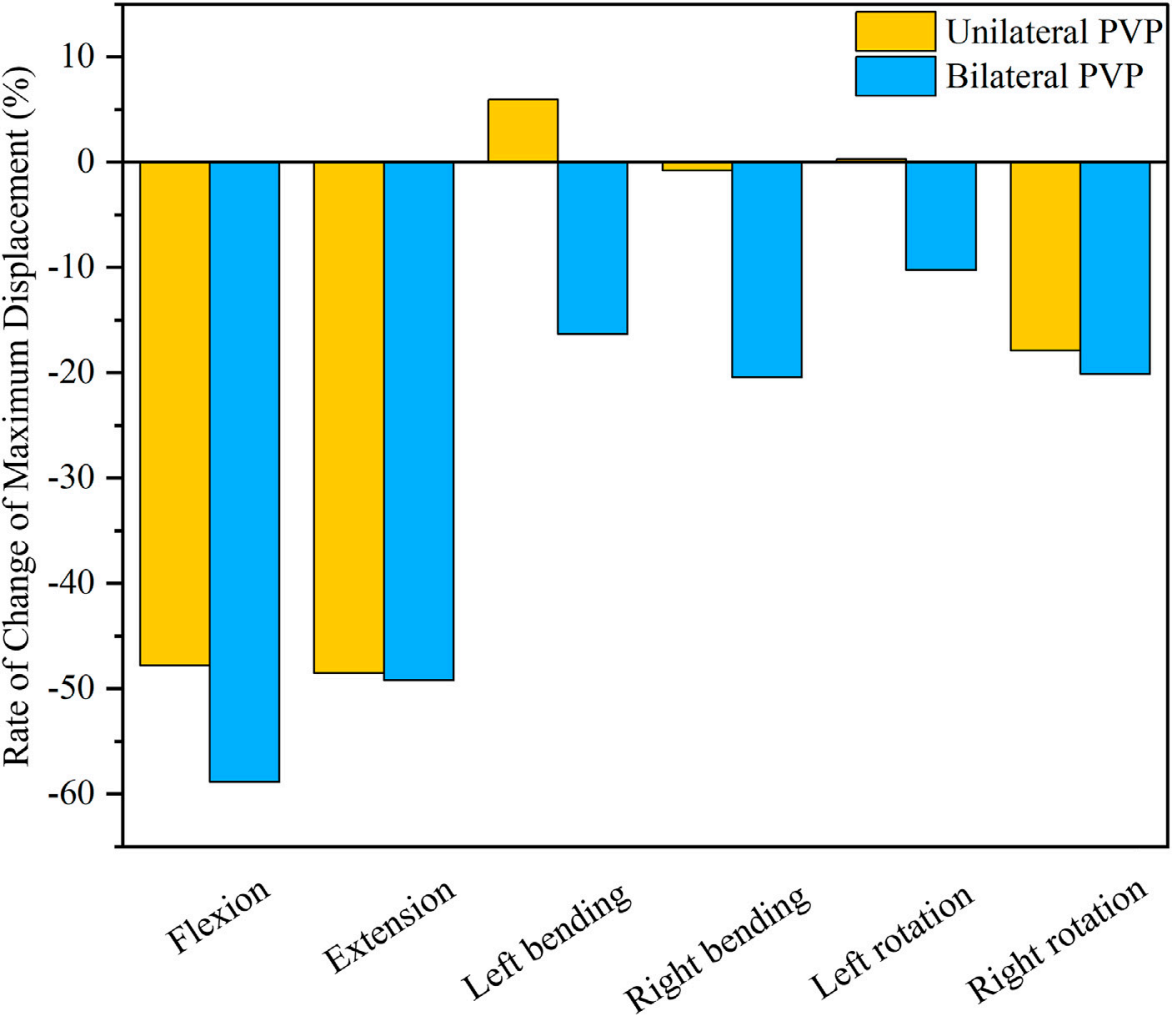
Rate of Change of Maximum Displacement of T12. Rate of Change of Maximum Displacement of T12 between UPVP and BPVP under flexion, extension, left/right bending, and left/right torsion.

Under flexion, extension, right lateral bending, and right axial rotation, the maximum displacement of T12 in UPVP decreased by 47.81%, 48.55%, 0.82%, and 17.90%, respectively, compared with the fractured model. Under left lateral bending and left axial rotation, the maximum displacement of T12 in UPVP increased by 5.60% and 0.27%, respectively, compared with the fractured model. However, the maximum displacement of T12 in BPVP decreased under all loading conditions and was lower than UPVP, which decreased by 58.88%, 49.24%, 16.31%, 20.42%, 10.27%, and 20.13% compared with the fractured model (Figure 11).

## Discussion

Complications of PVP include recompression of the PVP-operated vertebrae, new fractures at the neighborhood vertebrae, and degenerative disc disease (DDD) (Al-Nakshabandi, 2011; Zhang et al., 2017; Nagaraja et al., 2013; Feng et al., 2018). All of these can affect the quality of life of patients who underwent PVP. Some researchers found that postoperative complications of PVP are closely related to biomechanics (Belkoff et al., 2001; Sun et al., 2011; Belkoff et al., 2000; Molloy et al., 2003). Therefore, in this study, different approaches to PVP were analyzed from the biomechanical perspective by the finite element method. It was found that there was a different biomechanical impact on the vertebral bodies and intervertebral disc, resulting in different incidences of postoperative complications. In the following discussion, the postoperative complications of PVP will be analyzed from a biomechanical perspective.

First, for the recompression of the PVP-operated vertebrae, some studies have shown that the distribution of bone cement and the stiffness of the vertebral body, which affect stress balance on the latter, are important influencing factors (Liebschner M. et al., 2001; Zhang et al., 2017; Chen B. et al., 2011). n this study, there was obvious stress concentration on the side of the cement injection of T12 in UPVP under six loading conditions. This indicated that UPVP causes the stiffness of only one side of the vertebral body to be improved with bone cement while the other side is still damaged cancellous bone with lower stiffness, suggesting that the asymmetric distribution of bone cement could cause the stress to concentrate on the augmented side of the vertebral body. This asymmetric distribution of bone cement might impair physical strength on the non-augmented side, leading to fracture recurrence (Yu et al., 2017; Lee et al., 2014). However, this study found that there was no obvious high-stress concentration region on the anterior and middle columns of the T12 vertebral body in BPVP compared with UPVP. This indicated that the symmetrical distribution of bone cement produced by the even injection of BPVP into the vertebral body could make the structure and stiffness of the vertebral body more symmetrical, which could produce a balanced stress distribution on the latter. In a retrospective study, Hou et al. (2018) found that the symmetrical distribution of cement in the vertebral body could significantly reduce the incidence of recompression. The symmetrical distribution of bone cement would provide better mechanical support for the injured vertebra and prevent the micro-movement of the fracture, which is not only beneficial for reducing the risk of the recompression of the PVP-operated vertebrae but also for relieving pain caused by recompression (Cotten et al., 1996; Liebschner M. A. K. et al., 2001).

According to the three-column theory by Denis, recompression of the vertebrae could be affected by the stability of the spine (Denis, 1983; Denis, 1984). The poor stability of the spine could lead to an increased risk of recompression. Maximum displacement has been proposed as a parameter of stability (Li Q. et al., 2013). In this study, BPVP could reduce the maximum displacement of T12 under all loading conditions and much lower than UPVP. This result indicated that BPVP could reduce the movement of the spine and was more effective than UPVP in recovering vertebral body stability, which was also demonstrated by Liebschner M. et al. (2001). Therefore, BPVP could also reduce the incidence of recompression through more satisfactory stability.

In conclusion, compared with UPVP, BPVP, with the symmetrical distribution of bone cement and superior stability, could reduce the incidence of the recompression of PVP-operated vertebrae and relieve pain caused by recompression.

Second, for the new fractures at the neighboring vertebrae, some studies have shown that load transfer, which could be along the longitudinal axis of the spine to the adjacent vertebral body, is one of the important causal factors affecting the fracture risk of the untreated adjacent vertebrae (Chiang et al., 2009; Baroud et al., 2003; Fahim et al., 2011; Polikeit et al., 2003c; Liebschner M. et al., 2001). Kim et al. (2010) suggested that the symmetrical distribution of bone cement in the vertebral body promotes a balanced load transfer. In this study, it was found that compared with the compression fracture (Pre-augmented), the peak stress on the adjacent T11 in BPVP was decreased under all conditions, while in UPVP it was increased under left/right lateral bending conditions. Also, the peak stress on the L1 of BPVP under all loading conditions was the least of the three models (including the compression fracture, UPVP, and BPVP). These results indicated that BPVP with the symmetrical distribution and stiffness of bone cement would produce a balanced load transfer, which could alleviate the stress on adjacent vertebral bodies and reduce the risk of new fractures. Therefore, BPVP has biomechanical advantages over UPVP in reducing the risk of adjacent vertebral fractures. Interestingly, this study also found that compared with UPVP, the peak stress of T11 in BPVP was lower under left/right lateral bending but higher under left/right axial rotation. This indicated that the effect of BPVP is more satisfactory than that of UPVP in lateral bending, and it might be helpful to avoid excessive spinal rotation for patients who underwent BPVP. From the above point of view, BPVP plays a certain role in reducing the incidence of new fractures at the neighborhood vertebrae.

Third, for DDD, Nagaraja et al. (2013) reported that vertebroplasty can cause increased compression of adjacent intervertebral discs in osteoporotic spines, which could lead to DDD. According to Feng et al. (2018), this is because vertebroplasty leads to impaired nutrient supply to the disc. Stress is one of the factors that affect the degeneration of the intervertebral disc (Spina et al., 2021). Our results showed that for the T12-L1 intervertebral disc, the peak stress of BPVP was less than UPVP and compression fracture (Pre-augmented). This suggested that compared with UPVP, BPVP might be helpful to reduce the incidence of the degeneration of the T12-L1 intervertebral disc, which could reduce the risk of DDD and improve the patient’s life quality. Compared with the compression fracture (Pre-augmented), the peak stress of the T12-L1 intervertebral disc in UPVP was increased under left/right lateral bending conditions. This is similar to the results of Elmasry et al. (2018). Although the loading conditions that occurred were different (the reason might be that the fracture model established is different), the results of Elmasry et al. and our study are consistent with Baroud et al. (2003), who reported that by injecting cement into the vertebral body, spinal loads were transferred between the superior and inferior discs, resulting in increased intradiscal pressure.

To sum up, this study found that compared with UPVP, BPVP with the symmetrical distribution of bone cement has certain advantages in restoring the stability of the spine and reducing the incidence of postoperative complications such as recompression of the PVP-operated vertebrae, new fractures at neighboring vertebrae, and DDD by better restoring the biomechanics of the spine. Therefore, BPVP can improve the quality of life of patients and bring satisfying long-term clinical effects.

The finite element model has several limitations in this study. First, some simplifications were necessary for creating the model, such as the characteristics of intervertebral discs, ligaments and paraspinal muscles and he assumptions of linear, isotropic, and homogeneous material properties for the finite element model. However, these simplifications might have an impact on stress and displacement. Elmasry et al. (2017) modeled the intervertebral disc as biphasic materials constituted of a solid matrix embedded in a fluid phase, which made the disc more realistic and yielded higher stress than the simplified disc in our study. Second, the spine model did not provide a detailed simulation of the osteoporotic trabecular bone micro-architecture and was assigned 67% of the elastic modulus of the healthy bone quality. Besides, the fracture was ideally performed as a planar cut, and boundary conditions were simplified compared to the complex *in vivo* loadings. All of these might have an impact on stress and displacement. Third, for simulating bone cement with a vertical cylinder, the similar method to simulate bone cement had been reported by Liang et al. (2015a), and they found that although different shapes of bone cement to simulate PVP could produce different stress and displacement, the same conclusion could be reached. Therefore, using a vertical cylinder to simulate bone cement not only leads to the same conclusion as using different shapes of bone cement but also reduces the amount of computer calculations and ensure the repeatability of the study. However, simplification of bone cement into a vertical cylinder still cannot reflect the irregular shape of the bone cement in reality and might have some effects on stress and displacement. Fourth, our finite element model was based on data from one person that could not be representative of all patients treated with vertebroplasty. Therefore, based on the current study and the general experiences with datasets of multiple individuals, future biomechanical analyses should establish finite element models that can more accurately reflect the human condition and formulate an appropriate vertebroplasty plan for individual patients. Furthermore, *in vitro* biomechanical experiments and clinical study to evaluate the findings from this study also would be conducted in the future.

## Conclusion

In this study, the biomechanical differences between BPVP and UPVP were compared *via* the finite element method to determine the relationship with complications. The results suggested that BPVP could balance the stress of the vertebral body, reduce the maximum stress of the intervertebral disc, and has advantages in stability compared with UPVP. Therefore, BPVP could reduce the incidence of postoperative complications, which could provide guidance for clinical application and promising clinical effects for patients.

## Data Availability

The raw data supporting the conclusion of this article will be made available by the authors, without undue reservation.
